# Patient-reported outcome measures in studies on hallux valgus surgery: what should be assessed

**DOI:** 10.1007/s00402-024-05523-y

**Published:** 2024-09-09

**Authors:** F. T. Spindler, S. Ettinger, D. Arbab, D. A. F. Scientific Committee, Christina Stukenborg-Colsman, Sabine Ochman, Stefan Rammelt, Hans Polzer, Natalia Gutteck, Norbert Harrasser, Christian Plaaß, S. F. Baumbach

**Affiliations:** 1 Department of Orthopaedics and Trauma Surgery, Musculoskeletal University Center Munich (MUM), University Hospital, LMU Munich, Ziemssenstraße 5, 80336 Munich, Germany; 2 University Hospital for Orthopaedics and Trauma Surgery, Pius-Hospital Oldenburg, Georgstrasse 12, 26121 Oldenburg, Germany; 3 Department of Orthopedic and Trauma Surgery, St. Elisabeth-Hospital Herten, Member Faculty of Health Witten/Herdecke University, Im Schlosspark 12, 45699 Herten, Germany; 4 Deutsche Assoziation für Fuß und Sprunggelenk e.V., Strasse des 17. Juni 106-108, 10623 Berlin, Germany

**Keywords:** Patient-reported outcomes measures, PROMs, Hallux valgus, Forefoot deformity, Surgery, Systematic review

## Abstract

**Introduction:**

In recent years, there has been an increasing demand for patient-reported outcome measures (PROMs) to assess the outcome following orthopedic surgery. But, we are lacking a standard set of PROMs to assess the outcome of hallux valgus surgery. The aim of this study was to analyze the chosen patient rated outcome scores used in studies reporting on hallux valgus surgery.

**Materials and methods:**

The study was based on a previously published living systematic review. Included were prospective, comparative studies of different surgical procedures or the same procedure for different degrees of deformity. Four common databases were searched for the last decade. Study selection, data extraction, and risk of bias assessment were made by two independent reviewers. Data assessed were the individual PROMs used to assess the outcome of hallux valgus surgery.

**Results:**

46 studies (30 RCTs and 16 non-randomized prospective studies) met the inclusion criteria. The most commonly used clinical outcome measures were the AOFAS (55%) and the VAS (30%). No differences were found between frequency of the individual scores per the level of evidence or the type of osteotomy.

**Conclusion:**

Based on a systematic literature review, the AOFAS and VAS are the most frequently used outcome tools in studies assessing the outcome following hallux valgus surgery. Based on the literature available, the MOXFQ is a more valid alternative.

**Level of evidence:**

Level I; systematic review of prospective comparative (level II) and randomized controlled trials (level I).

## Introduction

Assessing the outcome following orthopedic surgery remains a hot topic of debate. In general, the outcome is frequently assessed by imaging, range of motion (ROM), or patient-reported outcome measures (PROMs). In recent years, there has been an increasing demand for PROMs, both from scientific committees and governments. This stays especially true for elective foot and ankle surgery, as it has to show its efficacy to both, the patient and the insurance provider.

In a previously published living systematic review, the authors assessed the outcome following hallux valgus surgery [1]. Despite a considerable number of eligible studies, all of which had a level of evidence of I or II, a meta-analysis could only be conducted for the HVA, IMA, and AOFAS. This dramatically highlights the grossly missing standardization of study protocols in foot and ankle surgery. In the initial study, the authors did not conduct a formal analysis of all the PROMs assessed in the different studies.

Therefore, the aim of the current study was to reanalyze the studies included in the living systematic review per the chosen patient rated outcome scores. The results were discussed to identify a possible standard set of outcome measures for hallux valgus outcome research.

## Materials and methods

### Study selection and data extraction

The study was based on a previously published living systematic review and is part of the current revision process of the German guidelines for hallux valgus surgery (033-018). The review was registered a priori (Prospero #CRD42021261490), conducted per the Preferred Reporting Items for Systematic Reviews and Meta-Analyses (PRISMA-P) guidelines [2] and the PICOS criteria [3]. Four common databases (Medline (PubMed), Scopus, Central and EMBASE) and the grey literature were searched from 01/01/2012 to 01/31/2023. Prospective studies comparing either two surgical procedures or one surgical procedure for different stages of hallux valgus deformity were included. The whole study selection-, data extraction- and assessment-process was conducted by two independent reviewers (SE, SFB). Disagreement at any stage was resolved by discussion with a third reviewer (HP).

### Data assessment

The level of evidence was rated per the recommendations of Wright et al. [4] and risk of bias was assessed by the Risk of Bias 2 (RoB 2) tool [5] or the Newcastle–Ottawa scale [6], where appropriate. The primary outcome parameter assessed was the PROMs used in each individual study. PROMs included the visual analog scale for pain (VAS), clinician-based outcome scores, and any quality-of-life (QOL) score. These were analyzed descriptively per their frequency, the level of evidence [4], the type of osteotomy performed, and the quality of the journal (i.e. impact factor) in which the study was published.

## Results

### Study selection

**Fig. 1:**
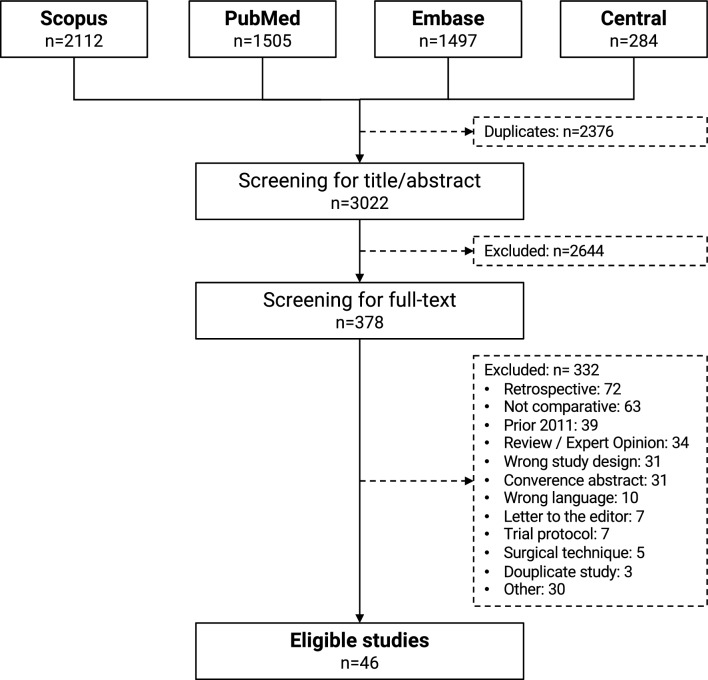
PRISMA flow chart. n: number of studies

Figure 1 depicts the study selection process. 3022 studies were screened for title and abstract and 378 for full-text. Finally, 46 primary studies [7–52] were enrolled for qualitative analysis consisting of 40 studies comparing different surgical procedures [7, 9–20, 22–26, 28, 30–33, 35–42, 44–52] and six studies comparing the same surgical procedure for different severities of HV deformity [8, 21, 27, 29, 34, 43]. 30 studies were RCTs (RoB2: 2 × high risk, 28 moderate risk) and 16 non-randomized comparative studies (Newcastle–Ottawa-Scale: 6 ± 1 points ≙ moderate risk).

Overall, only eight different outcome measures were used in the studies. The individual PROMs per the studies’ level of evidence, in descending order, and per the surgical technique are outened in Table 1. The two most used clinical outcome measures were the AOFAS (55%) and the VAS (30%). The remaining six PROMs were used in less than 5% of the studies each. No correlation could be found between the frequency of the individual scores and the level of evidence or the type of osteotomy. Also, the usage of PROMs per the various journals and the corresponding impact factors of the publishing journals did not differ (Table 2).

**Table 1:** Outline of the included PROMs per level of evidence and the type of osteotomy

Outcome score	Total (%)	Level of evidence		Type of osteotomy						
		Lvl I	Lvl II	Chevron distal	Scarf	Chevron proximal	Arthrodesis	MIS 2. Gen	MIS 3. Gen	Other
Studies (n) [Total / Lvl of Evidence] Study arms (n) [Osteotomies]	46 (100%)	36 (78%)	10 (22%)	31	23	3	5	8	9	18
AOFAS	38 (55%)	25 (52%)	13 (62%)	27 (40%)	12 (40%)	3 (60%)	5 (56%)	6 (55%)	9 (56%)	16 (59%)
VAS	21 (30%)	15 (31%)	6 (29%)	9 (23%)	10 (33%)	1 (20%)	4 (44%)	3 (27%)	7 (44%)	6 (22%)
SF-36	3 (4%)	3 (6%)	0	1 (3%)	1 (3%)	1 (20%)	0	0	0	1 (4%)
MOxFQ	3 (4%)	2 (4%)	1 (5%)	1 (3%)	4 (13%)	0	0	0	0	1 (4%)
ACFAS	1 (1%)	1 (2%)	0	0	1 (3%)	0	0	0	0	1 (4%)
Kitaoka-MTP1-Score	1 (1%)	1 (2%)	0	2 (5%)	2 (7%)	0	0	0	0	0
FAOS	1 (1%)	1 (2%)	0	0	0	0	0	2 (18%)	0	0
Roles and Maudsley	1 (1%)	0	1 (5%)	0	0	0	0	0	0	2 (7%)

*n* number, *Lvl* level of evidence [4], *AOFAS* American Orthopaedic Foot and Ankle Society Score, *VAS* visual analogue scale, *SF-36* Short Form-36, *MOXFQ* Manchester-Oxford Foot Questionnaire, *ACFAS* American College of Foot and Ankle Surgeons scoring scale, *MTP1* first metatarsophalangeal joint, *FAOS* Foot and Ankle Outcome Score

**Table 2:** Outline of the included PROMs per the different journals in descending order per the impact factors

Outcome score	J Orthop Translat	J Bone Joint Surg Am	Bone Joint J	BioMed Central	Clin Orthop Relat Res	Int Orthop	Foot Ankle Int	J Orthop Surg Res	Medicina (Kaunas)	Foot Ankle Surg	Arch Orthop Trauma Surg	In Vivo	Orthop Surg	J Foot Ankle Surg	Acta Orthop Traumatol Turc	Indian J Orthop	Z Orthop Unfall	J Am Podiatr Med Assoc	Foot Ankle Spec	Acta Orthop Belg	Clin Ter	Eur Res J
Studies (n)	1	4	3	1	1	2	8	1	1	5	2	1	1	6	2	1	1	1	1	1	1	1
IF (2022)	6,6	5,3	4,6	4,3	4,2	2,7	2,7	2,6	2,6	2,5	2,3	2,3	2,1	1,3	1,0	1,0	1,0	0,7	0,6	0,4	0,4	0,3
AOFAS		4	3	1	1	2	7	1	1	3	2	1	1	5	2	1	1	1			1	1
VAS		3	1	1		2	5		1	3	1	1			1			1				1
SF-36		1					1													1		
MOxFQ							1			1									1			
ACFAS										1												
Kitaoka-MTP1-Score																				1		
FAOS	1																					
Roles and Maudsley														1								

*IF* impact factor, *n* number, *Lvl* level of evidence [4], *AOFAS* American Orthopaedic Foot and Ankle Society Score, *VAS* visual analogue scale, *SF-36* Short Form-36, *MOXFQ* Manchester-Oxford Foot Questionnaire, *ACFAS* American College of Foot and Ankle Surgeons scoring scale, *MTP1* first metatarsophalangeal joint, *FAOS* Foot and Ankle Outcome Score

## Discussion

The AOFAS and VAS scores were the most frequently applied assessments to rate the outcome in hallux valgus surgery for the past eleven years. This result was independent of the studies’ level of evidence, the type of surgery performed, or the impact factor of the journal in which the study was published.

The current study is part of the revision process of the German Hallux Valgus guideline. The authors’ intention was to define a standard set of PROMs to evaluate the patient-rated outcome of hallux valgus surgery. This set of scores should be valid and allow a comparison to current literature. Our approach was to re-analyze the studies identified in a previously published living systematic review which included prospective studies, published after 2012, comparing either two surgical procedures or one surgical procedure for different stages of hallux valgus deformity. Consequently, the analyzed data set could have a selection bias. Still, only higher quality studies were included and one could assume, that these studies spend the most time on properly designing the methodology used. Although eight different scores were used, the by far most frequently assessed ones were the AOFAS and VAS. The VAS was scored on a ten-item Likert scale in all cases.

Throughout foot and ankle literature, the AOFAS Clinical Rating Systems [53] are the most commonly used outcome score. This stayed true for the current analysis on studies on hallux valgus surgery. Due to the fact that this study is a component of the revision of the German Hallux Valgus guidelines, one limitation of our study is that only studies published after 2012 were included. The large number of studies included, however, enables for further interpretation of the state of the art of PROMs used in hallux valgus surgery outcome studies.

Although the AOFAS remain the top dog, they have been criticized for several reasons. First, the AOFAS are not PROMs, as they combine a patient rated and clinician rated section. They are clinician-based outcome measures, which evaluate patients’ pain, function, and alignment based upon clinicians’ observations. They therefore do not eliminate a possible observer bias [54]. Subsequent studies demonstrated their limitations and the American Orthopaedic Foot and Ankle Society does not endorse the scales due to insufficient reliability and validity [55]. Guyton et al. [56] conducted a Monte Carlo computer modelling technique to assess limitations of the AOFAS scoring system. They simulated for each item the responses of different, idealized patient populations. The two major points of concern per the reliability of the AOFAS score were: the scoring items are used as absolute descriptors (e.g. “no limitation” or “no pain”) and are therefore susceptible for an interpretation bias by both, patients and clinicians; the limited number of response intervals leads to a pronounced floor- and/or ceiling effect [56, 57]. Furthermore, the AOFAS overemphasis the symptoms pain, equaling a maximum of 40 points, resulting in inferior outcome measures concerning other symptoms like stiffness or deformity [58]. Finally, the MCID is less for older patients compared to younger patients, and those patients with middle-range disability generally have less MCID values compared to those with minimal or severe disability [59]. Use of the AOFAS Clinical Rating Systems as the sole instrument is therefore discouraged [55].

Due to these limitations, the foot and ankle community, should strive to establish a new standard to assess patient rated outcomes, not only in hallux valgus surgery. There are more than 89 assessment tools available which measure overall foot and ankle function, overall health, or are designed for specific diagnoses and procedures.

General quality of life outcomes scores or pain scores are not enough to evaluate a hallux valgus population. Other measures have been developed and tested for a wide variety of pathologies in foot and ankle surgery (FFI, FAAM, AAOS, FHSQ and others). Furthermore, a disease-specific outcome measure is necessary to assess outcomes. MOXFQ, SEFAS and FAOS have been evaluated for hallux valgus surgery.

The FAOS [60] consisting of five subscales, with 42 items was derived from the Knee injury and Osteoarthritis Outcome score (KOOS) [61]. It was validated on patients with general foot and ankle disorders first, then on patients with hallux valgus deformity [54]. It showed acceptable validity, reliability, responsiveness, and comparability to the SF-36 in four out of five subscales [54]. The sports and recreation subscale showed little responsiveness to hallux valgus surgery and ceiling effects were present for the activities of the daily living and sports scale. The symptoms subscale showed a low correlation to the SF-36 due to the foot-specific items assessed in the FAOS [54].

The SEFAS consisting of 12 items, with 3 subscales was developed for assessing ankle replacement surgery but has been tested on a hallux valgus population with good psychometric properties [62]. It presented good validity, reliability, and responsiveness with a lack of MCID data.

The MOXFQ, consisting of 16 items with 3 domains, has been validated for foot and ankle disorders in general and specifically for a hallux valgus population. It has been extensively tested and was more sensitive than general health measures for quantifying hallux valgus surgery [63, 64].It has been compared to other outcome measures with good results. Comparison of the MOXFQ and the SEFAS demonstrated good psychometric properties with excellent test–retest reliability and internal consistency for both scores with superior responsiveness for the MOXFQ [65]. The MOXFQ showed higher responsiveness to detect changes over time or after surgery and has been translated and evaluated in more languages than SEFAS [65].

Recently the EFAS score has been validated in a population of hallux valgus patients with a short follow-up time of 6 months [66]. It has been tested with fair construct validity and reliability. However, responsiveness has not been evaluated at all. Further validation and comparative studies are necessary to rate the EFAS score in comparison to the above-mentioned PROMs.

Based on these considerations, it is even more surprising, that the vast majority of authors still relies on the AOFAS as their primary outcome score. Only three studies assessed the MOXFQ and no studies the SEFAS or EFAS score. The expert panel revising the German guidelines for hallux valgus surgery therefore recommends the use of the MOXFQ as the primary outcome score, due to its higher responsiveness and availability in more languages. There is a strong recommendation to also assess the VAS (10-item Likert scale). The use of the EFAS will be reconsidered in the next guideline revision. The AOFAS should only be assessed as a secondary outcome parameter to allow a higher comparability between the different studies.

## Conclusion

Based on a systematic literature review, the AOFAS and VAS are the most frequently used outcome tools in studies assessing the outcome following hallux valgus surgery. Based on the literature available, the MOXFQ is a more valid alternative.
